# Fluorine-18-Labeled Fluorescent Dyes for Dual-Mode Molecular Imaging

**DOI:** 10.3390/molecules25246042

**Published:** 2020-12-21

**Authors:** Maxime Munch, Benjamin H. Rotstein, Gilles Ulrich

**Affiliations:** 1 University of Ottawa Heart Institute, Ottawa, ON K1Y 4W7, Canada; benjamin.rotstein@uottawa.ca; 2 Department of Biochemistry, Microbiology and Immunology, University of Ottawa, Ottawa, ON K1H 8M5, Canada; 3 Institut de Chimie et Procédés pour l’Énergie, l’Environnement et la Santé (ICPEES), UMR CNRS 7515, École Européenne de Chimie, Polymères et Matériaux (ECPM), 25 rue Becquerel, CEDEX 02, 67087 Strasbourg, France; gulrich@unistra.fr

**Keywords:** positron emission tomography, Fluorine-18, fluorescence, molecular imaging, dual-mode imaging, radiofluorination

## Abstract

Recent progress realized in the development of optical imaging (OPI) probes and devices has made this technique more and more affordable for imaging studies and fluorescence-guided surgery procedures. However, this imaging modality still suffers from a low depth of penetration, thus limiting its use to shallow tissues or endoscopy-based procedures. In contrast, positron emission tomography (PET) presents a high depth of penetration and the resulting signal is less attenuated, allowing for imaging in-depth tissues. Thus, association of these imaging techniques has the potential to push back the limits of each single modality. Recently, several research groups have been involved in the development of radiolabeled fluorophores with the aim of affording dual-mode PET/OPI probes used in preclinical imaging studies of diverse pathological conditions such as cancer, Alzheimer’s disease, or cardiovascular diseases. Among all the available PET-active radionuclides, ^18^F stands out as the most widely used for clinical imaging thanks to its advantageous characteristics (t_1/2_ = 109.77 min; 97% β^+^ emitter). This review focuses on the recent efforts in the synthesis and radiofluorination of fluorescent scaffolds such as 4,4-difluoro-4-bora-diazaindacenes (BODIPYs), cyanines, and xanthene derivatives and their use in preclinical imaging studies using both PET and OPI technologies.

## 1. Introduction

Observation of cellular and molecular processes in vitro or in vivo is of tremendous interest in biomedical sciences. Molecular imaging allows for identification and quantification of biological processes occurring at the molecular level and therefore is an important tool for researchers and clinicians [1]. The use of a chemical probe displaying selectivity toward a particular target enables the investigation of precise phenomena such as receptor/protein expression, oxidative stress, enzymatic activity, and changes in cellular environment [2,3,4,5]. Translation of molecular imaging to clinical science has proven useful to diagnose, prognose, and monitor disease progression; select treatment strategies; predict or evaluate treatment efficiency; and guide surgeons during operations [6,7,8,9,10].

Common molecular imaging techniques include positron emission tomography (PET), single-photon emission computed tomography (SPECT), magnetic resonance imaging (MRI), and optical imaging (OPI; including fluorescence imaging and bioluminescence imaging) [11]. Although researchers have made great progress both in the elaboration of higher specificity and sensitivity probes and in the development of improved acquisition systems, these techniques still show limitations in spatial/temporal resolution or sensitivity. Thus, the accuracy required for medical diagnosis is not always reached by using only one imaging technique. During the last 15 years, a surge in the design of multimodal imaging technologies (e.g., PET/MRI, PET/OPI) has been observed [12]. This approach combines the advantages of two or more imaging modalities, allowing one’s advantages to compensate another’s limitations. To facilitate the use of multimodal imaging, single probes detectable via several modalities were developed. Multi-responsive probes are advantageous compared to a mix of several single-function probes as potentially only one injection is required; a reduced amount of time is needed to acquire information; and, finally, the pharmacokinetics and toxicity of a single probe remain constant compared to a mixture of probes, which can complicate clinical translation [13].

In vivo or in vitro OPI consists of detecting a luminescent probe emitting photons in the UV/visible (350–750 nm) to the near infrared (NIR; 750–1500 nm) ranges upon excitation with a higher energy light source [13]. Currently used luminescent probes include fluorescent proteins, organic fluorescent dyes, lanthanide coordination complexes, nanoparticles, and quantum dots [5,14,15,16,17]. For biomedical imaging applications, desirable emissions are usually located in the NIR region where autofluorescence from biomolecules such as hemoglobin and oxyhemoglobin are at the lowest [18]. As a result, improved signal to background ratio, tissue penetration and reduced photobleaching, light attenuation, and scattering are obtained compared to probes emitting in the UV/visible range. The use of a lower energy light is also beneficial as it is less destructive for tissues, thus decreasing eventual side effects and the invasive character of an imaging procedure. In the same fashion, two-photon active fluorescent probes are currently gaining interest [19]. Advantages of OPI lie in the low cost of this technique as both probes and imaging facilities are quite affordable compared to PET or MRI [11]. Chemical engineering to tune optical and biological properties of probes can bestow unique features such as activation, environmental sensitivity, dual-emissive probes, and multiplex imaging [20,21,22,23]. However, OPI clinical translation is still limited to fluorescence-guided surgery due to low tissue penetration depth of photons in this energy range. In comparison to other modalities (PET for instance), OPI also suffers from lower sensitivity, leading to a lower spatial resolution for in-depth tissues and demanding injection of higher doses, possibly inviting unwanted toxicity [11]. Despite these arguments, OPI is a suitable candidate for multimodal imaging in combination with another imaging modality, such as PET, which is not limited by penetration depth.

PET imaging relies on the injection of a probe bearing an unstable radioisotope that can be localized by detection of two antiparallel high-energy gamma photons generated when positrons emitted by the decaying radionuclide annihilate with surrounding electrons from the medium [24]. This method allows in vivo 3D imaging of biological processes with a spatial resolution up to the millimeter scale and a sensitivity around the subnanomolar range [11]. Some common positron emitters used for PET include ^11^C (t_1/2_ = 20.4 min), ^13^N (t_1/2_ = 10.0 min), ^18^F (t_1/2_ = 109.8 min), ^68^Ga (t_1/2_ = 67.6 min), and ^89^Zr (t_1/2_ = 78.4 h), which are produced using either a cyclotron or a generator depending of the radionuclide, followed by chemical incorporation in the compounds of interest usually denoted as radiotracer or radiopharmaceutical [24]. The scope of this review only includes ^18^F-based PET radiotracers displaying fluorescence properties [25,26,27,28,29,30,31,32]. The diversity of available radionuclides allows labeling of biologically active species including small molecules ([^15^O]H_2_O, [^13^N]NH_3_, [^18^F]NaF, etc.) [33,34,35], small proteins ligands (inhibitors, enzyme substrates, etc.) [6,36,37], antibodies (mainly by conjugation with radiometal chelates) [38], or nanoparticles [39]. PET’s most important advantage consists in its superior depth of penetration, allowing for the study of deep tissues. Moreover, PET scanners allow time-course detection and quantification of the radiotracer throughout the body, providing accurate data about radiotracer pharmacokinetics and physiological changes [24]. However, the presence of a high-cost cyclotron or generator nearby is required, thus limiting the use of PET to specific sites. In addition, depending on the utilized radionuclide, the radiotracer’s half-life is a limiting criterion as the production, injection, and data recording must be performed within a specific period. Moreover, an efficient radiolabeling method affording the radiotracer in high radiochemical purity (RCP; usually close to 99%), high molar activity (A_m_; above 1 Ci/μmol), and in a decent amount of time (up to three half-lives in general) may be required.

**Figure 1:**
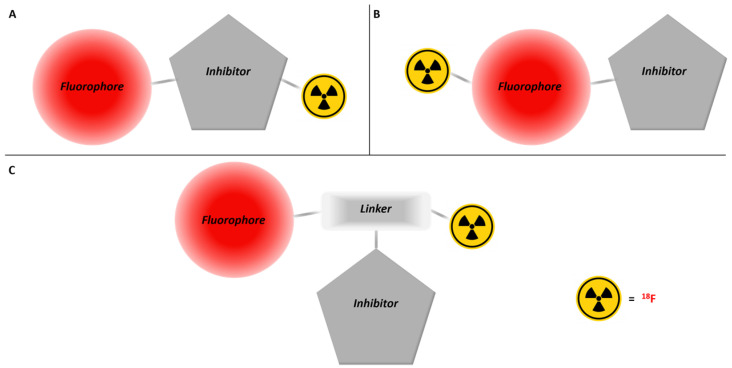
Schematic representations of the three main ways to link the different parts of a bimodal ^18^F-labeled positron emission tomography (PET)/optical imaging (OPI) probe. (**A**) Fluorescent and radioactive tags are both attached directly on the bioactive molecule at two different locations. (**B**) The fluorescent tag is used as a radiolabeling site and is linked to the bioactive molecule. (**C**) The fluorescent tag, the radionuclide and the bioactive molecule are all linked together through a linker.

Given the strengths and limitations of these modalities, several research teams recently focused their work on the development of bimodal PET/OPI active probes [13,40]. Three main synthetic strategies have been identified thus far. The first consists in tagging a biomolecule with a fluorescent dye and a radiolabeled prosthetic group at two different positions (Figure 1A). This method requires sequential labeling and is mostly used for peptides and antibodies. In addition, long-lived isotopes are preferred due to the rather extended lifetimes and slower pharmacokinetics of these kinds of molecules in vivo compared to smaller ligands [41]. The two other methods refer to the radiolabeling of the fluorescent dye either directly on the fluorophore scaffold (Figure 1B) or by using a linker (Figure 1C) [42,43]. In this case, shorter-lived isotopes are also practical, especially ^18^F, as it is often present in bioactive molecules and its small size minimizes perturbation of pharmacokinetic properties. Although its short half-life might appear problematic, its potential for high molar activity, the numerous ways to introduce it, and the metabolic stability of the C–F and B–F bonds make ^18^F a suitable candidate for the development of PET/OPI dual-mode imaging probes [44,45]. A few examples of ^18^F-labeled nanoparticles showing utility for OPI have also been described [46,47]. This field is, however, out of the scope of this review [48].

This literature review focuses on the recent developments in the synthesis of ^18^F-labeled small fluorescent dyes and their applications in molecular imaging. Synthetic approaches are discussed for each type of organic fluorescent dye followed by their applications in preclinical imaging. A recent review by Klenner et al. also discusses multimodality imaging with ^18^F [49]. The current work differs in its greater focus on alternative fluorophores, small molecules, and demonstrated applications in molecular imaging.

## 2. ^18^F-Labeled Fluorescent Dyes

### 2.1. BODIPYs

**Scheme 1:**
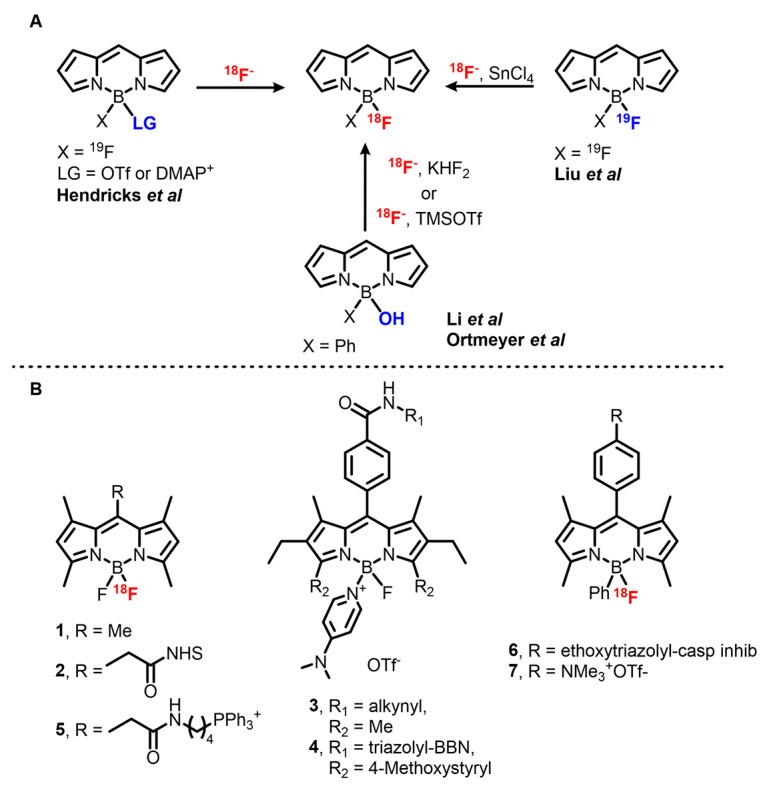
(**A**) Major radiosynthetic methods used for ^18^F-4,4-difluoro-4-bora-diazaindacene (BODIPY) radiofluorination. Corresponding references: Triflate or DMAP substitution [59]. Hydroxyl group substitution [42,60]. Isotopic exchange [61]. (**B**) Examples of bimodal PET/OPI probes based on ^18^F-BODIPY scaffolds.

Since their first report in 1968 by Treibs and Kreuzer, 4,4-difluoro-4-bora-diazaindacenes (BODIPYs) have been thoroughly studied and are now considered to be one of the most important families of organic fluorophores [50]. Locking of the cyanine monomethine backbone by the boron atom confers to BODIPYs excellent (photo)stability, high extinction coefficients (ε), high quantum yields (Φ_F_) in solution, sharp absorption bands, and sharp emission bands, positioning them as a dye of choice for many applications [51,52]. Moreover, possible NIRF emission and global neutrality of these dyes are of particular interest for imaging purposes. One drawback of BODIPY dyes is their low Stokes shifts (usually below 1000 cm^−1^), reducing signal-to-background ratio due to reabsorption and use of narrow filters. Nowadays, biomedical applications of BODIPY dyes are mainly focused on cellular imaging and sensing, however, examples of NIRF OPI and photoacoustic imaging (PAI) have been described [53,54]. Given the importance of BODIPY dyes as fluorescent probes, several groups have been involved in the development of dual-mode imaging agents including OPI-PET, OPI-SPECT, or OPI-MRI [53,55,56]. Recently, Ali et al. published an overview of radiolabeled BODIPYs in the literature including ^125^I, ^111^In, and ^89^Zr adducts [57]. The majority of ^18^F-BODIPYs are labeled on the boron center (Scheme 1), however, the first example reported by Perrin et al. describes radiolabeling via the use of a boronic ester [58]. This work is one of the first of a series on boronic ester-mediated ^18^F radiolabeling. This method is more often used for the radiosynthesis of xanthene- and cyanine-based tracers and is further detailed in the related sections (vide infra). Other methods rely on the presence of the central boron atom and can be classified into three types, one based on two or three steps labeling through an activated intermediate and the others based either on hydroxy substitution or on direct isotopic exchange (IEX), both acid-catalyzed.

Development on the first method started in 2008 when Hudnall and Gabbaï described a BODIPY-4-dimethylaminopyridine (DMAP) cation for turn-on sensing of fluoride by displacement of DMAP from the boron atom [62]. This procedure was adapted for preparative purposes in 2012 by Hendricks et al. and afforded [^18^F]BODIPY **1** with a 68 ± 23% d.c. RCY [59]. However, the high temperature required for the fluoride–DMAP exchange is incompatible with sensitive moieties, especially bioconjugation handles such as *N*-hydroxysuccinimide (NHS) esters. Moreover, upon extended heating or in the presence of acid, product decomposition and exchange of ^18^F with released ^19^F can occur, decreasing the yield and molar activity of the product. This issue was addressed by a one-pot radiolabeling procedure through activation of [^19^F]BODIPY **1** with TMSOTf and direct exchange with ^18^F in presence of TfOH. The two reactions occur quickly at 20 °C and the intermediate can be stabilized in solution by buffering the media with a mild, non-nucleophilic base such as DIPEA or 2,6-lutidine. This modified method afforded [^18^F]**1** within 2 min in 67% RCY, with A_m_ = 0.96 Ci/μmol. Following a slightly modified method, NHS ester [^18^F]**2** was obtained and subsequently coupled with the monoclonal antibody trastuzumab with a 19.9% d.c. RCY. Soon after, Yuan et al. used this method to synthesize OPI/PET probes for mitochondria imaging by coupling [^18^F]**1** with an amine bearing triphenylphosphonium derivative to afford [^18^F]**5** with A_m_ = 0.37 mCi/μmol [63].

In 2014, Weissleder’s group investigated a one-step version of this reaction by sequentially adding Tf_2_O, *t*BuOH, and a BF_2_-BODIPY precursor to the ^18^F/*n*-tetrabutyl ammonium bromide (TBAB) mix [64]. A wide range of commercially available BODIPY-NHS esters and PARPi-FL, a PARP1 inhibitor currently studied for detection of epithelial cancers, were labeled with this method [65]. However, despite the efforts invested in the study of this reaction, the presence of unreacted starting material in the reaction media limited A_m_ values to a range of 5–80 mCi/μmol for radiolabeling of [^18^F]**1** and therefore dampened the use of this procedure for radiopharmaceutical production.

In 2014, Brizet et al. used the BODIPY-DMAP stability to synthesize a series of PET imaging precursors [66]. They showed that the DMAP adduct withstands Huisgen cycloaddition conditions, isothiocyanate synthesis, and their subsequent coupling with amines. Green emitting BODIPY-DMAP **3** bearing a terminal alkyne was successfully coupled with peptides such as bombesin, and the DMAP part was successfully displaced using KF, suggesting that BODIPYs could be used as prosthetic groups for late-stage peptide radiolabeling. However, a major side product of this reaction is the BF(OH) complex stemming from hydrolysis of the DMAP group. 

Simultaneously with the development of this first method, Gabbaï et al. described an acid-mediated substitution of a hydroxy group attached to the central boron atom of the BODIPY core with fluorine by treatment of a B(Ph)(OH)BODIPY with KHF_2_ in THF [67]. This method was then modified to allow ^18^F labeling in aqueous conditions. [^18^F]BODIPY **7** was obtained in 22 ± 3% d.c. RCY and a A_m_ > 1.4 Ci/μmol without preliminary ^18^F drying step by reaction of the precursor with KHF_2_ in a MeOH/[^18^O]H_2_O mix at 70 °C for 10 min [42]. Ortmeyer et al. used a slightly simplified method to label an isatin-based caspase inhibitor [^18^F]**6** in 11% ± 6.1% d.c. RCY in 91 ± 6 min with a high molar activity (A_m_ > 4.48 Ci/μmol) [60]. 

Finally, Lewis acid-promoted IEX on BODIPY dyes, developed in 2013 by Gabbaï et al., is the most used reaction for ^18^F labeling of this family of dyes [61], possibly due to ready implementation and the ability to afford an extended range of tracers in high yields. Among the set of Lewis acids used, SnCl_4_ in excess (8 to 14 molar excess in this case) was shown to be the most effective, affording [^18^F]**7** with a nearly quantitative yield (95% d.c. RCY) in ACN at room temperature and in only 10 min. This method is compatible with NHS esters, making it attractive for the synthesis of imaging probes. Other bioconjugation groups are tolerated as has been demonstrated for terminal alkynes and azide moieties. In addition, BODIPY conjugates with small peptides such as RGD, an α_V_β_3_ integrin receptor ligand used for cancer imaging, can be obtained in very good RCY (82%). In addition, BODIPYs bearing various substituents such as alkyls, aryls, and styryls afforded OPI/PET probes with emission bands between 500 and 650 nm. The downside of this method resides in the low molar activity of the products, which are usually in the mCi/μmol range. This is likely due to the presence of remaining starting material sharing the exact same structure as the product in the reaction mixture and thus being impossible to remove. However, it appears that the use of [^18^F]BODIPYs afforded via this method has not been hindered. Indeed, this method was applied to the synthesis of [^18^F]BODIPY radiotracers bearing various functional groups, peptides (RGD, RGD dimers, bombesin, MT-MMP1 substrates), different small molecule-based inhibitors (PARP1, PACMA31), and lipids either at late stage or before conjugation [55,68,69,70,71,72,73].

### 2.2. Fluoresceins and Rhodamines

**Scheme 2:**
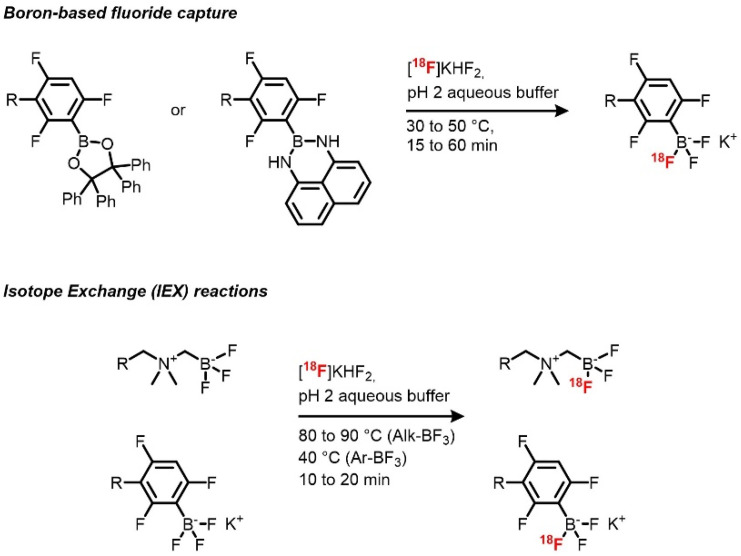
Radiofluorination of boron-based prosthetic groups.

Similar to BODIPYs and cyanines, xanthene derivatives including fluorescein and rhodamine are remarkable fluorophores with high Φ_F_ and ε, and good photostability [74,75]. Compared to the former families, their major difference resides in the presence of an equilibrium (influenced by solvent and pH) between the closed spiro-lactone form, which is non-emissive, and the open emissive form, allowing development of environmentally sensitive probes. Regarding OPI, alkylated rhodamine derivatives are usually preferred as their emission band is red-shifted compared to fluoresceins (λ_em_ around 570 nm and 510 nm, respectively) and they present an improved photostability [74]. Despite extensive work on fluorescein and rhodamine synthesis and functionalization, emission maxima above 700 nm are rarely described, which can be a disadvantage when compared to previous dyes. However, these dyes are well suited for dual mode PET/OPI probes as their phenyl moiety represent a good platform for introduction of ^18^F by use of a prosthetic group. In the case of xanthene derivatives, these groups include mainly [^18^F]fluoroethyl tosylate derivatives and boron trifluoride derivatives (Scheme 2).

The first examples of ^18^F-labeled rhodamine were described by Packard et al., who synthesized [^18^F]fluoroethyl rhodamine-B via nucleophilic substitution on [^18^F]fluoroethyl tosylate by the rhodamine’s carboxylic acid group [76,77]. This two-step procedure afforded the probe with a 35% d.c. RCY in 90 min with A_m_ = 35 mCi/μmol. Purification of the intermediate [^18^F]fluoroethyl tosylate allowed A_m_ to reach 67 mCi/μmol. Soon after, the authors also studied the effect of the prosthetic group on the pharmacokinetics of rhodamine-B by replacing the fluoroethyl group by fluoropropyl, fluoro di-, and fluoro tri-ethyleneglycol groups [78]. Lower A_m_ was observed due to side reactions with remaining ditosylate precursors. This methodology was also applied to rhodamine-4Me, -6G, and -101 in similar RCY and A_m_ [79]. Finally, replacement of the one-pot two-steps procedure by the synthesis of the tosylated diethylene glycol ester of rhodamine-6G readily labeled with ^18^F afforded the corresponding tracer with A_m_ up to 7 Ci/μmol [80]. RCYs were unfortunately decreased (6 ± 3% d.c.) due to ineffective elution of ^18^F ions from the QMA cartridge and partial retention of the lipophilic tracer on the synthesizer’s tubing. However, this method represents an easy way to afford ^18^F-rhodamines with high molar activities.

**Scheme 3:**
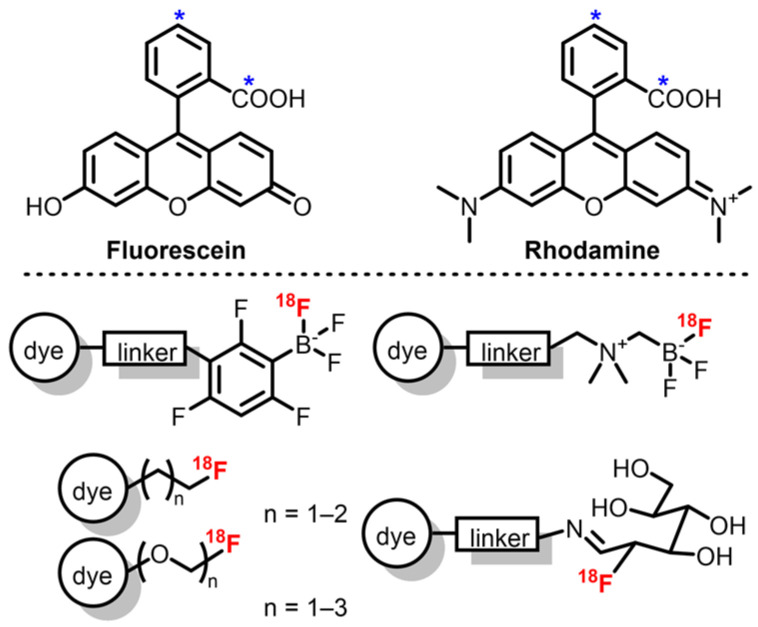
General structure of fluorescein and rhodamine dyes (blue stars * indicate positions used for prosthetic group linkage) and structures of described prosthetic groups used in xanthene dyes radiofluorination.

^18^F boron trifluoride xanthene derivatives can be achieved by fluoride capture by a boron-containing reactive group and by IEX on boron trifluoride species (Scheme 3) [81,82]. The use of inorganic fluoride captors has been applied to radiochemistry in order to allow late-stage labeling of biomolecules with high molar activities, and boron-based prosthetic groups have been used in the design of several prosthetic groups [83,84]. Radiosynthesis using the IEX reactions has been previously demonstrated on organic molecules, then followed by silicon- and boron-based prosthetic groups. Boron trifluoride derivatives as radiolabeling precursors of fluorescent compounds were first described by the team of Perrin. These groups proved to be useful for late-stage radiolabeling thanks to the high rates of exchange and the slightly acidic reaction conditions, allowing their use on a wide range of compounds. In the case of xanthene dyes, [^18^F]sulforhodamine B was quickly (10–15 min of reaction time) afforded with d.c. RCYs between 45 and 75%, high RCPs, and very high A_m_ (up to 14.3 Ci/μmol), allowed also by the use of a low amount of precursor [81]. Other advantages of this method are the absence of ^18^F drying step and prolonged heating (40 °C), which respectively shortens the synthesis and potentially allows the application of this method to a broad scope. The only drawback of this method resides in the slight instability of aryl boron trifluorides that are partially hydrolyzed during the reaction, giving rise to the formation of the corresponding boronic acid. However, in this case, the side product is well defined from the targeted one and can be easily removed by preparative HPLC.

Sulforhodamine 6G was radiolabeled on an alkyl trifluoroborate group, in a similar way [85]. The corresponding dual mode probe was obtained with a 25% d.c. RCY in 25 min and a A_m_ of 4 Ci/μmol. A trivalent version of the prosthetic group was also developed to synthesize probes bearing one sulforhodamine 6G and two RGD peptides with RCYs from 11% to 33% and A_m_ up to 3.95 Ci/μmol [85,86]. Up to now, only one fluorescein derivative has been synthesized by Ting’s group, who used the same technology [87,88]. A fluorescein derivative bearing an azide group was coupled to alkyl BF_3_ moiety by Huisgen cycloaddition to afford the radiolabeling precursor. IEX reaction using similar conditions was then performed to obtain the corresponding PET/OPI dual probe with a 57% RCY in around 60 min and A_m_ 0.4 Ci/μmol from 116 mCi of [^18^F]fluoride. 

[^18^F]Sulforhodamine B was also afforded by fluoride capture [82]. Instead of a one-step radiofluorination from an arylboronic ester, a one-pot two-step starting from an arylborimidine bearing a terminal alkyne was chosen. Radiofluorination of the prosthetic group was performed by reacting ^18^F fluoride in acidic condition (pH = 2) in both non-carrier-added (NCA) and near-NCA conditions followed by a cycloaddition reaction to the azido-sulforhodamine B. In NCA conditions, the target compound was obtained with a 15% RCY and a very high A_m_ of 15 Ci/μmol, and near NCA conditions afforded a higher 30% RCY while allowing a lower but still high A_m_ of 7.5 Ci/μmol from around 20 mCi each (after trapping and concentration). Compared to the IEX-based methods, high molar activities are here allowed by the stoichiometry of the reaction, enabling the introduction of 3 F atoms, thus theoretically multiplying the A_m_ values by 3. Again, aqueous medium, mild conditions (30 °C), short reaction time (15 min), and use of non-dried ^18^F fluoride are quite appreciable conditions for radiolabeling of biomolecules. 

Finally, AlJammaz et al. described the radiolabeling of a rhodamine conjugated with [^18^F]2-fluoro-2-deoxyglucose ([^18^F]FDG) [89]. Radiosynthesis was performed quite easily by heating the precursor with [^18^F]FDG in acetate buffer at 60 °C for 10 min. Reaction of the hydroxylamine with the aldehyde of the opened form of [^18^F]FDG afforded the aldoxime conjugate with a nearly quantitative RCY (starting from [^18^F]FDG) in around 20 min and a A_m_ of 70 mCi/μmol. 

Xanthene derivatives proved to be a suitable platform for radiofluorination, affording dual mode PET/OPI compounds in good yields and high molar activities via various methods. However, emission maxima of current radiolabeled compounds do not exceed 590 nm, which could appear as a drawback compared to red-shifted dye such as Cy7. Moreover, radiolabeling sites are currently restricted to the central group, which could be a limitation in terms of synthetic approaches. Nevertheless, rhodamines’ outstanding optical properties make of them a dye of choice for the development of PET/OPI dual mode probes.

### 2.3. Cyanines

**Scheme 4:**
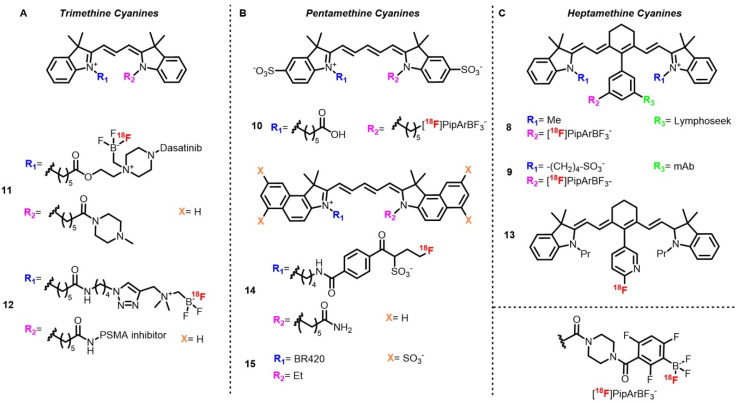
Structures of described ^18^F-labeled trimethine (**A**), pentamethine (**B**), and heptamethine (**C**) cyanine dyes.

Cyanines are among the most used fluorophores along with BODIPYs and xanthene derivatives, especially for biomedical applications. First synthesized in 1856 by Williams, several types of cyanines have now been developed, including classical cyanines, hemicyanines, squaraines, merocyanines, and oxonols [90,91,92]. To our knowledge, only classical cyanines have been labeled with ^18^F for dual-mode OPI-PET imaging. From a photophysical point of view, these dyes have several advantages such as high extinction coefficients and quantum yields in solution and can offer a red-shifted emission up to 1000 nm [91,93]. Cyanines display low toxicity and therefore are commonly used in biomedical applications. Notably, indocyanine green (ICG) and fluorescein are among the only fluorescent indicators approved by the Food and Drug Administration (FDA) and European Medical Agency (EMA), and IRDye800 CW is the subject of several clinical trials [13,94,95]. Possible functionalization on terminal nitrogen atoms and on the central methine group is favored for introduction of bioconjugation links and radiolabeling sites. The weaknesses of cyanines for molecular imaging are mainly due to their low Δ_SS_ and their poor photostability, causing reabsorption and photo-bleaching, respectively. Since 2010, numerous ways of ^18^F radiolabeling cyanines have been described (Scheme 4). Four pathways can be differentiated: late-stage fluorination by IEX, fluoride capture by boronic esters or aromatic nucleophilic substitution, and two-step labeling by introduction of a pre-labeled prosthetic group. 

The first example describes ^18^F labeling of a heptamethine cyanine (Cy7) conjugated with Lymphoseek (**8**), a dextran functionalized with mannose and diethylenetriamine penta-acetic acid (DTPA), used for lymph node imaging in melanoma or breast cancer [43]. The method used relies on fluoride capture by a boronic ester in acidic aqueous conditions to afford a radiolabeled trifluoroborate [58]. Although this method requires carrier-added conditions (KHF_2_) and does not always allow separation of the radiolabeled trifluoroborate and the boronic ester, the first studies on this method described an A_m_ (d.c.) of 0.12 Ci/μmol from 100 mCi of starting activity. Radiolabeling of the Cy7–Lymphoseek conjugate following an optimized method afforded the target compound with RCY up to 3% and A_m_ values of 0.242 Ci/μmol. This procedure is quite appreciable for biomolecule radiolabeling as it is carried out in aqueous solutions and at low temperature. Moreover, starting activity is used as received at the end of the beam without a drying step. In this case, low A_m_ did not prevent imaging experiments, as the authors determined that A_m_ from 1 to 50 Ci/mmol was sufficient. Ting’s group also described a late stage radiolabeling of Cy7–mAb conjugate (**9**) (cetuximab) by adaptation of this method to solid phase synthesis [96]. The authors took advantage of the presence of the dioxaborolane group to introduce a biotin handle used for immobilization on an agarose-streptavidin gel. Once treated with [^18^F]fluoride, the target radiotracer, which was no longer bound the gel and possessed A_m_ = 0.16 Ci/μmol after purification by SEC-HPLC, was able to be injected directly after elution. The same reaction was utilized for a ^18^F N-functionalized pentamethine sulfo-cyanine pentamethine (Cy5) with a free carboxylic acid (**10**) moiety for tumor xenograft imaging [97]. 

IEX was also described on cyanine-based dual mode probes by Ting and coworkers, who labeled a trimethine cyanine (Cy3) functionalized on one of the nitrogen atoms with dasatinib (**11**), a kinase inhibitor used for glioma treatment and imaging [98]. Last step radiofluorination was then performed by heating the precursor with [^18^F]fluoride in acidic conditions. Attractively, this method is simple, rapid, and withstands water, as no quaternary methyl ammonium (QMA) trapping and azeotropic drying are required. Purification is performed by reverse phase HPLC, with the probe being obtained in 62% d.c. RCY with A_m_ = 0.38 Ci/μmol in 26 min. The same method was used to label a sulfocyanine Cy3–PSMA conjugate (**12**) where the alkyl BF_3_ group was attached on one terminal nitrogen and PSMA on the other nitrogen atom [99,100]. The probe was obtained in lesser yield but comparable A_m_, likely due to the heating (10–15 min at 80–90 °C). This tracer was purified by slow elution over a C18 cartridge with the help of a syringe pump. The authors emphasized the importance of the slow flow rate (40 mL/h) to avoid coelution with unreacted [^18^F]fluoride.

Recently, Valliant et al. described introduction of ^18^F directly at the Cy scaffold by aromatic nucleophilic (S_N_Ar) substitution on a 2-nitropyridine group attached on the central methine position (**13**) [101]. In this example, the precursor used was a reduced cyanine (hydrocyanine) used as a turn-on optical probe upon oxidation for reactive oxygen species (ROS) imaging. The target tracer was obtained by usual S_N_Ar conditions by heating in presence of [^18^F]KF and K_222_ in basic polar conditions followed by preparative HPLC purification. If this method works quite well in this case, late-stage nucleophilic substitutions are usually limited to small molecules labeling or performed on prosthetic groups prior to bioconjugation due to the prolonged heating required. 

Indirect radiolabeling of cyanine dyes have also been described. The first example was published by Priem et al., who developed a hydrophilic prosthetic group on the basis of the ring opening of a propanesultone with fluoride and bearing a conjugation moiety [102]. Preparation of the prosthetic group was performed in three steps in 75 min, with an overall RCY of 20–30% d.c. before ligation to Cy5.5 via an NHS ester (**14**). Although the synthesis of the prosthetic group seems tedious and long, it represents a potential way to modulate the pharmacokinetics of small radiotracers by improving their hydrophilicities thanks to the sulfonic acid group. Moreover, very quick coupling reaction (1 min) and purification (SPE) shortens the overall synthesis. Another way to tune the pharmacokinetic of cyanine-based dual-mode tracers has been described by Schwegmann et al., who used mono-, bis‑, and tetra-sulfonated Cy5 coupled to BR420 (**15**), a barbiturate-derived inhibitor, to favor renal over hepato-biliary clearance [103]. Radiolabeling was performed by click chemistry ligation of [^18^F]**1**-azido-2-fluoroethane and the set of radiotracers were obtained in 104 to 138 min, with RCY between 9.7 and 19.2% d.c. and A_m_ ranging from 0.03 to 0.97 Ci/μmol. 

In the view of dual mode PET/OPI probes, the major advantage of Cy dyes is the facile access of near-infrared emitting dyes, as commercially available Cy dyes can emit up to 800 nm. However, as Schwegmann et al. observed, non-hydrophilic Cy dyes are quickly cleared via the hepatobiliary system and might need to be replaced with sulfo-cyanines. 

### 2.4. Curcumins

Curcumin is the major pigment of turmeric roots and a component of eastern traditional medicine and gastronomy [104]. It has long been studied for its biological and photophysical properties as it displays anti-cancer and neurological activities, among others [105,106]. As a fluorophore, curcumin exhibits only modest characteristics with absorption and emission bands located around 420 and 500 nm, respectively; Φ_F_ up to 17% in low polarity media; and fluorescence lifetimes below the nanosecond [104]. Moreover, its bioavailability is rather low due to poor hydrolytic stability [104,107]. However, its interesting biological properties have still encouraged researchers to radiolabel curcumin derivatives for amyloid plaques and cancer imaging. Moreover, complexation to a metal center and engineering on curcumin scaffold were performed in order to improve its properties and allow its use as a PET/OPI dual mode probe [107,108,109]. Radiofluorination of curcumin derivatives is mostly performed on the phenol groups by introduction of a ^18^F fluoroalkyl or fluoroethyleneglycol chain. It can also be labeled on the polymethine chain after modification of the β-diketone moiety.

**Scheme 5:**
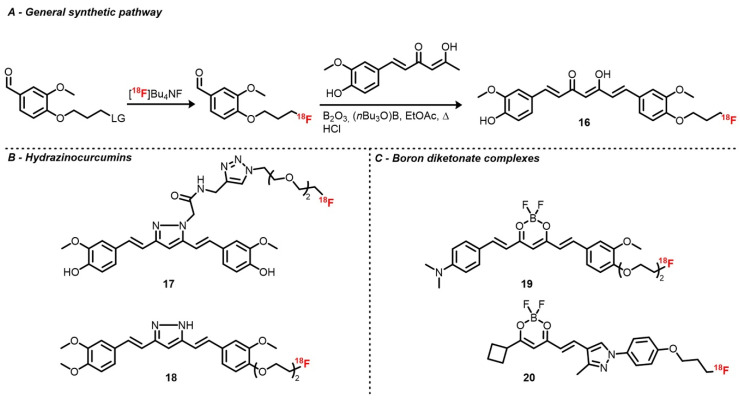
(**A**) General radiosynthetic pathway of classical ^18^F-curcumin derivatives. Structures of radiofluorinated boron diketonate analogs (**B**) and hydrazinocurcumins (**C**).

Ryu et al. investigated two curcuminoid radiofluorination methods [110]. First, relying on the classical nucleophilic substitution of a tosylate by [^18^F]fluoride on a curcumin derivative functionalized with a propyltosylate group on one of the two phenols, the corresponding [^18^F]fluoropropylcurcumin (**16**) was afforded with poor RCY and A_m_, likely due to curcumin instability. As an alternative, [^18^F]4-fluoropropyl-2-methoxybenzaldehyde was synthesized the same way prior to an aldolization step (105 °C for 1 min in EtOAc) with the boron diketonate complex, which was eventually hydrolyzed (Scheme 5). After HPLC purification, [^18^F]fluoropropylcurcumin was obtained with d.c. RCYs of 16–25% and A_m_ = 1.01 Ci/μmol. As brain uptake of this tracer was slightly lower than expected, an analog where the second hydroxy group was replaced by a methoxy was synthesized in order to improve its lipophilicity. The fluoropropyl chain was also replaced by a fluoroethyl one [111]. Along similar lines, curcumin derivatives with a pyrazole ring instead of the 1,3-diketone were developed. Rokka et al. described the radiolabeling of a pyrazole curcumin analog bearing a linker with a terminal alkyne aimed for prosthetic group introduction via cycloaddition [112]. This precursor was radiofluorinated with a one-pot two-step procedure by heating [^18^F]potassium fluoride–Kryptofix complex with azido diethyleneglycol tosylate in ACN, followed by cycloaddition at room temperature in the presence of copper sulfate. The radiotracer (**17**) was obtained with an overall RCY of 21 ± 11% and a very high A_m_, which was above 27 Ci/μmol. Shin et al. also described the radiofluorination of hydrazinocurcumin (**18**) analog with a diethyleneglycol tosylate chain on one of the two hydroxy groups [113]. Protection of the remaining hydroxy function and of the pyrazole NH group with methoxy methyl ether (MOM) was necessary to afford proper yields. Radiotracers were then obtained by classical substitution followed by acidic deprotection of MOM groups with d.c. RCY from 25 to 35% and A_m_ around 1.3 Ci/μmol. 

Recently, ^18^F boron diketonate complexes were also described following the one-pot two-step method initially described by Ryu et al. [114,115]. Almost simultaneously, the groups of Seong Choe and Ran published radiofluorinated BF_2_ complexes of curcumin (**19**) and half-curcumin derivatives (**20**), respectively. Both probes were inspired by the CRANAD family, a group of fluorescent dyes based on the BF_2_ diketonate scaffold [108]. Tracers **19** and **20** were obtained with d.c. RCYs around 20% and 51% and A_m_ of 1.16 and 1.19 Ci/μmol, respectively. During the development of these tracers, Kim et al. investigated the acid-mediated IEX radiofluorination in a similar manner as for BODIPYs, however, it was unsuccessful and only decomposition occurred [114]. Recently, Ting’s group patented a radiofluorinated curcumin derivative labeled via IEX using SnCl_4_ [116]. 

Although curcumin-based probes display limited optical and biological properties compared to the aforementioned scaffolds, the work described thus far paves the way for the development of PET/OPI probes, especially for amyloid plaque imaging. Efforts to improve curcumin’s in vivo stability should also be maintained in order to reach clinical translation.

### 2.5. Others

Aside from BODIPYs, cyanines, and xanthenes, many other fluorescent scaffolds have been labeled with ^18^F. However, use of their optical properties is mostly restricted to cell imaging.

In some cases, fluorescent scaffolds are introduced not in light of optical imaging experiments, but rather because they display good affinities with biological targets. This case is well illustrated by coumarins, which are common fluorophores. Up to now, ^18^F-labeled coumarins have only been used for their affinities with diverse targets such as dopamine D4 receptor and carbonic anhydrase IX [117,118,119,120]. ^18^F was introduced either via substitution on an alkyl tosylate chain or by S_N_Ar on prosthetic groups affording tracer in low to moderate RCYs and good A_m_. The absence of optical studies is likely due to the modest optical properties of the native backbone (blue centered absorption and emission, low Φ_F_) compared to fluorophores discussed above. However, after suitable functionalization, i.e., extension of the π-delocalized path or introduction of an electron-donating group, development of dual-mode PET/OPI probes could be considered [121].

A few examples of radiofluorinated porphyrinoids have also been described. Most recent syntheses use either classical direct tosylate substitution or click ligation with the precursor in decent yields and molar activities [122,123,124]. Kavali et al. also described condensation of [^18^F]*p*-fluorobenzaldehyde with pyrrole and *p*-methoxybenzaldehyde or on the corresponding tetrapyrrane precursor [125]. Although existing works focus mostly on radiosynthesis and preclinical evaluation based on PET and cellular uptake, use of the optical properties of porphyrin derivatives in OPI and especially photodynamic therapy (PDT) are promising. However, compared to their radiometalated analogs, ^18^F-porphyrinoids are still seldomly used, probably due to the shorter half-life of ^18^F, which sets time limitations in terms of synthesis and analysis. Moreover, introduction of ^18^F requires design of the precursor as it contains a labeling function that is not required in radiometallated porphyrins. 

Well-represented ^18^F tracers displaying fluorescence properties include azo dyes, stilbenes, phenazine derivatives, and 2-(aryl)azoles or benzazoles [126,127]. These scaffolds are often met in brain imaging projects but their optical properties (low ε, Φ_F_ in solution) often limit their use to conventional PET tracer evaluation and tissue section imaging. This is also the case for compounds that are not part of usual dye families [128,129].

^18^F-labeled analogs of common staining dyes have also been described. Indeed, acridine orange, ethidium, anthracene, and Evans blue analogs have been radiofluorinated, but only [^18^F]ethidium optical properties were used in a cell imaging experiment despite their red-centered emission [130,131,132,133,134].

## 3. Applications

### 3.1. Tumor Imaging

The main application of dual mode PET/OPI probes until now has consisted of tumor imaging. Indeed, PET/OPI imaging is well suited for cancer applications, as it can be applied from cell to whole-body imaging with only one compound, and molar activity requirements can be forgiving due to high target expression. In 2010, Ting and coworkers described the use of a [^18^F]Cy7–Lymphoseek conjugate for lymph node dual-imaging. Lymph nodes as well as lymph tracks were successfully identified by both modalities [43]. In addition, fluorescence imaging was used to guide node excision and for histology. This work nicely demonstrated the potential of PET/OPI dual imaging, especially in the case of lymph node imaging, which currently requires injection of a mix of two compounds [135].

Radiolabeled dyes alone, conjugates with small molecule inhibitors, short peptides, and antibodies have been used to target tumors. Indeed, the structure of some dyes confers them affinity with cancer cells, as is known for curcumin derivatives [105]. Using a radiofluorinated hydrazinocurcumin derivative, Shin et al. were able to image a glioma xenograft model in mice 30 min after injection using PET [113]. Although hydrazinocurcumins tend to show improved potency compared to regular curcumins, which is the reason of their use in this study, their fluorescence emission is centered in the blue region, reducing their use in OPI. In parallel, the recent emergence of [^18^F]BF_2_-curcuminoids displaying red-shifted emission and higher fluorescence quantum yields compared to classical curcumins might encourage tumor dual mode imaging with this family of dyes [114,115]. An and coworkers also described the preferential accumulation of an unconjugated ^18^F-labeled sulfoCy5 in an A549 xenograft tumor model (adenocarcinoma model) [97]. Tumor was clearly identified by PET 6 h post injection and biodistribution was performed using both modalities. The possibility to perform semi-quantitative biodistribution using fluorescence imaging represents a significant advantage for groups without PET facilities. Dye concentrations above 0.01 μM is required to observe difference in fluorescence intensity by fluorescence-activated cell sorting (FACS), implying that non-radioactive equivalent should be added to the tracer to reach a proper optical signal, thus diminishing molar activity. Conjugates with small inhibitors were also used as described by Carlucci et al. and Wang et al., who respectively deployed [^18^F]BODIPY FL-PARP1 and [^18^F]Cy3-dasatinib to image glioma models [72,98]. In the latter study, PET/OPI imaging was also used to investigate the accuracy of the convection-enhanced delivery (CED) of the tracer, with CED being an alternative delivery method, bypassing the blood–brain barrier (BBB). In both studies, data obtained by PET were correlated with OPI. 

Replacing small inhibitors by peptides enables development of PET/OPI probes with activated fluorescence properties. This strategy was used by Kondo et al. to study MT-MMP1 activity in a fibrocarcinoma model [71]. Indeed, by introducing a dark quencher in a MT-MMP1 substrate labeled with a ^18^F BODIPY, the authors observed selective turn-on fluorescence in MT-MMP1-expressing tumors, and no emission in MT-MMP1-free tumors was observed, with preferential uptake of the tracer also being confirmed by PET. Tumor xenografts were imaged as well using RGD peptide conjugates. Liu and coworkers used a [^18^F]BODIPY-RGD probe to image a glioblastoma xenograft model in nude mice. Differences in signal intensities between ex vivo imaging of harvested organs (notably liver, kidneys, and tumor) by PET and fluorescence was observed and assigned to various degrees of vascularization. Indeed, a higher concentration in hemoglobin or oxyhemoglobin attenuated the fluorescence signal but did not affect the PET signal [61].

Using their tripodal prosthetic group (vide supra), Perrin’s group synthesized a ^18^F-rhodamine–*bis*(RGD) conjugate used for in vivo PET followed by ex vivo fluorescence imaging of relevant organs. In their discussion, the authors mentioned the necessity of finely tuning the molar activity of the tracer [86]. As PET tracers with high molar activity are usually injected in very low doses (3 Ci/μmol, 30.0 pmol in this case), fluorescence signal intensity is too low to be observed. Although fluorescence intensity can be enhanced by dilution of the tracer with its non-radiolabeled analog, its uptake in the target is then decreased due to self-blocking. If possible, an optimal molar activity allowing observation of both signals should be determined. This could potentially be done by performing a set of experiments with various dilutions of the tracer with its isotopologue while taking the target density into account in order to determine a molar activity value and a dose allowing imaging with both facilities.

**Figure 2:**
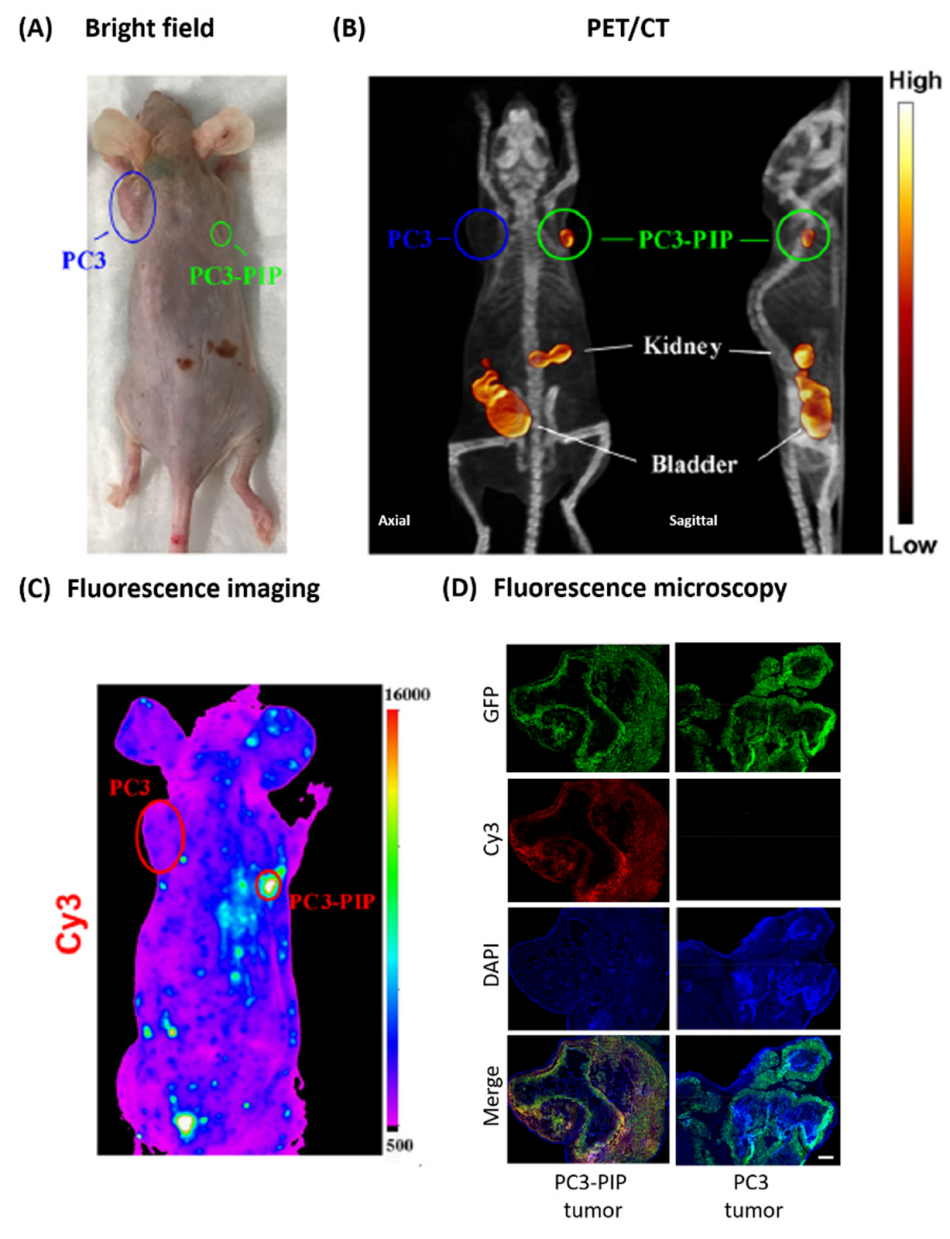
Selective PET and fluorescence imaging of PSMA-expressing (PC3-PIP) tumors compared to non-PSMA-expressing tumors (PC3) on a xenografted mouse model with PSMA-targeting probe [^18^F]**12**. (**A**) Bright-field image. (**B**) PET/CT imaging 6 h post-injection. (**C**) Whole-body fluorescence imaging of compound **12** showing selective accumulation in PSMA-expressing tumor. (**D**) Fluorescence microscopy of tumor sections. Compound **12** was selectively accumulated in PC3-PIP tumors (left column) as opposed to PC3 tumors (right column). Scale bar is 250 μm. Adapted with permission from [100]. Copyright 2019 American Chemical Society.

Prostate tumor models were also imaged by Ting’s group (Figure 2) [99,100]. Using fluorescence-guided resection, no tumor regrowth was observed compared to resection performed under bright light. The efficacy of fluorescence-guided resection has also been observed in several clinical trials, underscoring the need for a single compound that could potentially be used for diagnostic, guided surgery and post-surgical assessment [136,137]. Another advantage of fluorescence in cancer management resides in the use of tracer’s emission for intraoperative frozen section histology, which consists in imaging resected tissue sections during surgery to assess if tumorous tissues are remaining [138]. 

Finally, tumor tissues were also targeted using antibodies as described by Rodriguez et al., who used a [^18^F]Cy7-cetuximab conjugate [96]. Although the short ^18^F half-life might have been problematic given the slow pharmacokinetics of antibodies, tumors were still distinguishable 4.5 h after injection. Moreover, the presence of Cy7 allowed tumor NIRF imaging up to 72 h post-injection.

### 3.2. Cardiac Imaging

Cardiac imaging using dual mode PET/OPI is mostly represented by myocardial perfusion imaging (MPI). Moreover, as myocardium cannot be imaged externally by fluorescence in vivo due to its location, fluorescence is then mostly used for in vitro cellular imaging or ex vivo imaging. One way to perform MPI consists of targeting mitochondrial membrane, e.g., with [^18^F]flurpiridaz, which is currently in phase 3 clinical trial, or rotenone derivatives [139]. By analogy with the MitoTracker dyes that have good affinities with negatively charged mitochondrial membrane, researchers have developed a series of lipophilic, cationic dual-mode probes, bearing either phosphonium or ammonium cations, or taking advantage of positively charged rhodamines. The first of such examples were described by Packard’s group in 2010, who used [^18^F]fluoroethylrhodamine B in rat [76,77]. Although preliminary results showed fast hydrolysis in mouse serum, suitable stability was observed in rat and human analogs. MicroPET imaging also showed high accumulation in myocardium, and cellular imaging confirmed localization into mitochondria. Following this work, the authors investigated the nature of rhodamine dye and prosthetic group [78,79]. The [^18^F]rhodamine 6G-bearing fluorodiethylene glycol group displayed the best properties compared to its other derivatives, namely, a fast and constant accumulation in the heart and a lower uptake in the liver, assuring good contrast. 

[^18^F]BODIPYs have also been investigated for MPI. Li’s team developed a series of ammonium BODIPYs in both mice and rats [68]. Preferential uptake in the heart and high heart-to-lung ratios were observed for most of the tracers, however, they displayed lower heart-to-liver ratios compared to [^18^F]rhodamine 6G. Cellular imaging showed internalization of the dye and distribution in the cytoplasm varying with K^+^ concentration, thus indicating that uptake may be driven by membrane polarization. A [^18^F]BODIPY tetraphenylphosphonium salt was also studied by Yuan et al. [63]. Colocalization with rhodamines in cells indicated uptake in mitochondria, and PET/CT imaging in a mouse model of myocardial infarction revealed perfusion deficits. Ex vivo fluorescence imaging of the myocardium also revealed perfusion deficits, which colocalized with signals obtained using fluorescent microspheres used to identify ischemic areas. Finally, Cowan et al. used [^18^F]rhodamine 6G-labeled mitochondria to study their distribution by PET after injection in a rabbit ischemic heart and to locate lesions [140]. Although use of the optical properties of OPI/PET probes is currently limited to cell imaging, recent progress in intra-operative cardiac fluorescence imaging in preclinical methods and in clinical trials offers new perspectives. Indeed, fluorescence imaging proved to be a useful modality in atherosclerosis plaque identification and in myocardial perfusion assessment in pig models, and also intraoperative optical imaging in human has been used in bypass graft assessment [141,142,143]. Thus, a PET/OPI probe could potentially be used in pre-operative PET imaging followed by OPI guided surgery.

### 3.3. Brain Imaging

Despite the large number of PET probes and fluorescent dyes used for detection of amyloid-β (Aβ) plaques, neurofibrillary tangles, or α-synuclein, only a few examples of dual mode probes labeled with ^18^F have been described thus far. Moreover, some of the Aβ ligands displaying fluorescent properties are not suitable for in vivo imaging either due to poor brightness or low emission wavelengths. Furthermore, due to the skull and biological tissues decreasing the fluorescence signal, brain OPI imaging is limited in vivo. ^18^F-labeled curcuminoids and their difluoroborate analogs have been investigated as PET ligands and used for brain section imaging. Notably, Yang et al. and Kim et al. respectively used [^18^F]CRANAD-101 (**20**) and [^18^F]CRANAD-2 (**19**) derivatives for brain imaging of APP/PS1 model mice [114,115]. [^18^F]**20** allowed for clear identification of Aβ plaques by fluorescence microscopy of brain sections and by in vivo two-photon microscopy. Uptake measured by PET imaging was also slightly higher in transgenic mice compared to wild type. [^18^F]**19** gave similar results in brain sections and has been used for in vivo and ex vivo optical imaging with an In Vivo Imaging System (IVIS) apparatus. However, discrepancies between PET and OPI uptakes in Balb/C nude mice were observed due to poor metabolic stability of the probe. 

Recently, Wang et al. also applied dual-mode PET/OPI imaging to intracranial hemorrhage in mice, as fast identification is crucial for patient outcomes [144]. Using [^18^F]Cy3 or [^18^F]Cy5 bearing an amine reactive succinimidyl ester, the authors labeled red blood cells (RBCs) before injection into model animals. Fluorescence microscopy clearly revealed labeling of RBCs with both tracers. Labeling was stable and no transfer to surrounding cells was observed even after 14 h of incubation. In vivo PET/CT imaging allowed visualization of the hemorrhage with a good contrast. The same group also described the use of a radiofluorinated fluorescein derivative ([^18^F]Fc-AMBF_3_) for cerebrospinal fluid (CSF) leak detection and shunt viability assessment [87,88]. PET images allowed accurate observation of CSF leaks and dynamic tracer distribution through lumboperitoneal shunts. Moreover, spontaneous repair of CSF leaks was observed by PET. Fluorescence imaging was used to study the probe clearance and shunt imaging, confirming that the probe was distributed in the CSF and not taken up by surrounding cells. In this case, dual-mode PET/OPI imaging appeared to be a powerful tool for CSF imaging as PET allowed accurate identification of CSF leaks and because fluorescence can be used for guided surgery or diagnostic by fluorescence detection in the nasal cavity.

### 3.4. Other Applications

Two recent studies aimed to image brown adipose tissue (BAT) using [^18^F]BODIPYs, as it is involved in lipid metabolism and glucose and insulin homeostasis regulation. Therefore, it has recently been a target in the development of therapeutic approaches for diabetes and obesity. The first study, performed by Paulus et al., introduced a triglyceride-coupled [^18^F]BODIPY ([^18^F]BDP-TG) in chylomicron-like particles to mimic biological conditions and target BAT [145]. PET imaging allowed identification of BAT in C57Bl/6 mice fasted at 4 °C, with these conditions triggering BAT activation. Fluorescence imaging was used to confirm incorporation in particles. In the second study, Wang et al. employed a [^18^F]ammonium-BODIPY-targeting mitochondria previously mentioned in MPI imaging [146]. Preferential accumulation in brown adipocytes was observed by fluorescence microscopy compared to white adipocytes (WAT). In both cases, BAT tracer uptake was higher in stimulated animals compared to control at 1 h post-injection. However, the second tracer appeared to be more selective for BAT over other tissues involved in lipid uptake in comparison to its triglyceride-conjugated analog. As [^18^F]ammonium-BODIPY appears to be useful for BAT quantification, [^18^F]BDP-TG can potentially be used for BAT-related lipid metabolism.

High reactive oxygen species (ROS) concentration imaging was also investigated. The groups of Mach and Valliant respectively described ^18^F-labeled dihydroethidium (DHE) and hydrocyanine heptamethine (HCy7) derivatives as ROS dual probes with NIR emission turn-on properties [101,133]. Both compounds showed higher reactivity with superoxide radicals compared to hydroxy radicals or hydrogen peroxide. The probes’ turn-on properties in the presence of ROS were confirmed in vitro by cell assay in PC3 (known for ROS generation) cells for HCy7 and EMT6 cells with ROS generated by doxorubicin treatment for DHE. In vivo PET imaging with [^18^F]DHE showed higher myocardial uptake in doxorubicin-treated mice compared to control or mice injected with its oxidized analog. [^18^F]HCy7 biodistribution was also different compared to [^18^F]Cy7 in C57Bl/6 mice, indicating that [^18^F]HCy7 does not oxidize in vivo in non-pathological mice.

## 4. Conclusions 

PET/OPI dual mode imaging can shed light on biological mechanisms and has the potential to assist clinicians once translated. Fluorescence provides higher spatial resolution using fluorescence microscopy or naked eye observation. A strong advantage of this modality relies on the fact that the probe can potentially be imaged several hours post-injection, well after ^18^F decay, thus limiting the rush to perform image recording. The high sensitivity of PET nicely compensates for the low tissue penetration of fluorescence but is limited to sites with access to a radiopharmacy infrastructure. Thus, the synergy between these two modalities gives access to an extended amount of information both on probes’ biological behavior and on physiological processes. From a probe development perspective, PET and OPI can be used in cell- and tissue-based assays, in in vivo or ex vivo imaging experiments, and for biodistribution studies (quantitative for PET). Additionally, PET is well suited to metabolism studies and gives access to dynamic imaging. From a clinical point of view, PET is a powerful technique for diagnosis and can be used for pre-surgical planning or post-surgery/treatment monitoring. As radioactivity is more challenging to accurately detect in the operating room, fluorescence is complementary with precise, real-time, and easily amenable guidance for clinicians during open surgery or laparoscopy. Pre- and in-surgery analysis such as biopsy, intraoperative section analysis, or fluid- or cell-based assays are achievable using fluorescence.

Even if injection of a single compound should ease clinical translation compared to the use of a mixture, broad application of PET/OPI imaging is still limited. Indeed, fluorescence detection is limited in vivo, with only a few FDA-approved dyes. Moreover, the cost of radionuclide production, PET scanners, and limited number of production facilities are the current challenges in the field. However, constant development of new radiofluorination procedures affording high molar activity tracers quickly gives access to off-site tracer shipping. Progress in the development of fluorescent organic dyes and nanomaterials in parallel with alternative imaging facilities (photoacoustic imaging, two-photon fluorescence imaging, RAMAN, etc.) are subject of a tremendous interest and should push the boundaries of in vivo optical imaging along with the development of enhanced detection setups. Dual-mode PET/OPI probes might also be useful in Cerenkov imaging as radioactive decay can act as a stimulus for fluorescence emission. Described works thus far have proven ^18^F-radiolabeled fluorescent dyes to be promising targets for dual-mode imaging. Such compounds and their translation to clinic is likely to gather great interest in the upcoming years.
